# Hybrid material of structural DNA with inorganic compound: synthesis, applications, and perspective

**DOI:** 10.1186/s40580-019-0211-4

**Published:** 2020-01-06

**Authors:** Seung Won Shin, Ji Soo Yuk, Sang Hun Chun, Yong Taik Lim, Soong Ho Um

**Affiliations:** 1 School of Chemical Engineering, Sungkyunkwan University, 2066 Seobu-ro, Jangan-gu, Suwon, Gyeonggi-do 16419 South Korea; 2 SKKU Advanced Institute of Nanotechnology (SAINT), Sungkyunkwan University, 2066 Seobu-ro, Jangan-gu, Suwon, Gyeonggi-do 16419 South Korea

**Keywords:** Hybrid nanomaterial, Structural DNA nanotechnology, Inorganic compound, Disease treatment and diagnosis

## Abstract

Owing to its precise manipulation in nanoscale, DNA as a genetic code becomes a promising and generic material in lots of nanotechnological outstanding exploitations. The nanoscale assembly of nucleic acids in aqueous solution has showed very remarkable capability that is not achievable from any other material resources. In the meantime, their striking role played by effective intracellular interactions have been identified, making these more attractive for a variety of biological applications. Lately, a number of interesting attempts have been made to augment their marvelous diagnostic and therapeutic capabilities, as being integrated with inorganic compounds involving gold, iron oxide, quantum dot, upconversion, etc. It was profoundly studied how structural DNA-inorganic hybrid materials have complemented with each other in a synergistic way for better-graded biological performances. Such hybrid materials consisting of both structural DNAs and inorganics are gradually receiving much attention as a practical and future-oriented material substitute. However, any special review articles highlighting the significant and innovative materials have yet to be published. At the first time, we here demonstrate novel hybrid complexes made of structural DNAs and inorganics for some practical applications.

## Introduction

With the advent of advanced and state-of-the-art detection techniques developed, an unsolved and mysterious biomolecular mechanism working on many living organisms have been clearly turned out [1, 2]. The interactive networks among biological substances such as phospholipids, nucleic acids, and proteins are now organizing the basic principle regarding the mechanism of living organisms. A nucleic acid, which is represented as DNA, RNA, and referred widely to the genome, is commonly regarded as a genetic information storage which is mostly significant for a routine cellular operation and furthermore for population sustainability. Accordingly, DNA’s deepen understanding may be a prerequisite in clarification of such organism metabolism [3–5]. To effectively scrutinize it, in general and in the most usages, complementary DNA sequences coupling specifically with targeted oligonucleotides in organisms have been designed and explored directly in some biological system which is in inquiry [6–8]. A number of methodologies for enhanced signaling of specific DNA hybridization have been ingeniously contrived, in which there are a simple fluorescent detection of antisense hybridizations and an elaborate kinetics of strand displacements [9, 10].

Since 1980s, Nadrian Seeman has firstly introduced a new idea about DNAs’ use for a generic material, well known to be a structural DNA nanotechnology in a technological view [11, 12]. With his indebted and innovative labors, DNA oligonucleotides, which were solely thought to be a genetic code for survival of all living beings in Earth, have been recognized gradually as a polymeric nanostructure with repetitive functional residues. Its uses have been exploited into several academic and even industrial fields, not just in a fundamental biology. Together with the fast development of structural transformations, structural DNAs have been assembled into different forms including X- or Y-shaped DNAs [13–17] and complicated DNA origami of RNA-templated structural DNAs interwoven internally [18, 19]. They are now put into availability for effective drug delivery, cell metabolic manipulation and so on [20–22]. By some challenges for increasing their magnitudes, structural DNAs are even becoming a colloidal material. While basic properties of most of colloidal materials depend heavily on a change of their own sizes, interestingly, a dimension of structural DNA nanomaterials are determined primarily by a difference of DNA sequences as well as the size of DNAs. It is claimed that DNA materials may be made simple in a desired form, with all sequences designed intentionally. The final product may be varied greatly depending on its use [23–27]. Hence, structural DNA nanomaterials are obviously distinguishable from other conservative materials. However, that is not the case that structural DNAs may be a sole independent constitute in a manner which could not be shuffled with other materials.

**Fig. 1:**
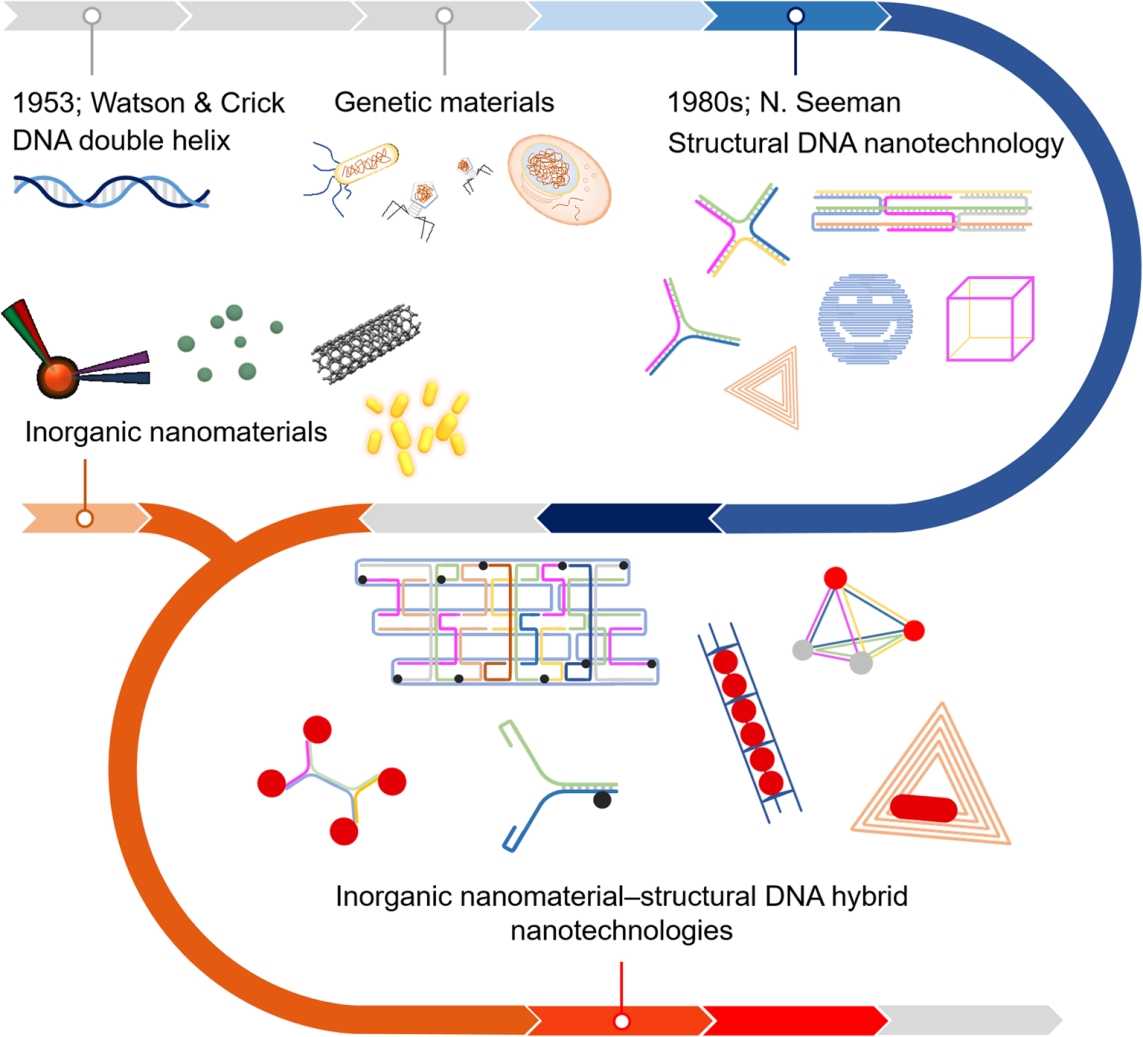
A representative scheme about the hybrid materials of structural DNA nanotechnology and inorganic compounds. The potential of hybrid complexes is more increasing in several practical applications including some biomedical issues of disease diagnosis and treatments. Their characteristic features have been complemented in a synergistic way. In this review, we introduce the up-to-date developments of the hybrid nanomaterials for some practical applications highlighted most recently

To date, there has been more than a hurdle to vault over in lots of biomedical issues involving a new diagnosis and disease treatment for countless diseases. It is strongly commanded that an ongoing biomedical system should take more than a function to effectively respond to some situations seriously broken. No single materials used nowadays, even in their progress, have performed all of things at a time. Thus, it is usually acknowledged that bright strategies on new material’s design is inevitably necessitated [28, 29]. Owing to the unique characteristics including a superior electromagnetics and an intrinsic biocompatibility, inorganic nanomaterials have been exploited a long while for biological uses. To apply them into cytosolic level, it has been issued to get them smaller [30–32]. As engaged with structural DNA nanomaterials, the detection power of inorganic compounds has been substantially boosted. A hybrid material made of structural DNAs and inorganics has received much attention as a future material substitute for some practical bioapplications. However, any special review articles highlighting a significant breakthrough of such materials science and engineering have yet to be published. Here we have demonstrated the up-to-date hybrid materials composed of structural DNAs and inorganic compounds at the first time and furthermore showed their progresses for many cutting-edge exploitations including some biological applications (Fig. 1). A type of nanoparticles used commonly as a building block and their characterizations are initially presented in the first section, successively followed by the second topic discussing their hybrid complexes and some related applications.

## Materials as a basic building block for hybrid complexation

Prior to more discussion about hybrid complexes, we will briefly introduce some representative basic materials used as a building-block in this subject. First, we discuss a principal feature and application of structural DNA nanotechnologies, followed by a variety of inorganic compounds that can be crossbred with DNA materials. Especially, significant contributions have been made with the benefit of a gold, iron oxide, quantum dot, upconversion nanoparticle, and carbon nanotube. Each nanomaterial has been defined well in its characters involving some physicochemical properties such as sizes and surface charges, and widely used for the strategic design of some functional nanocomplexes for a few of practical applications.

### Structural DNA nanotechnology

**Fig. 2:**
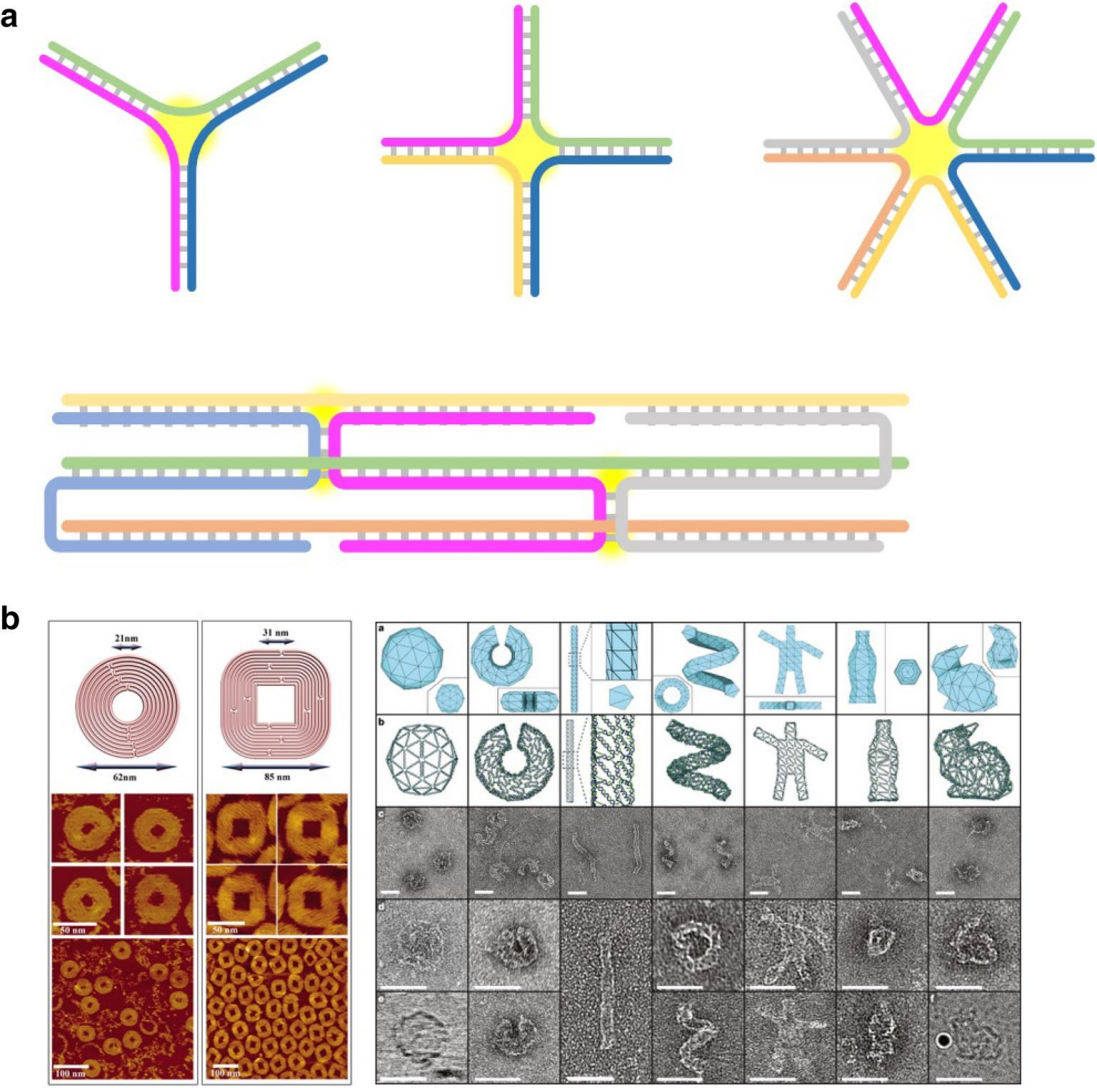
**a** Basic building blocks of structural DNA nanomaterials. Junctional motif, which is formed by a selective complementary hybridization, serves as a basic building block of structural DNA nanomaterials. **b** Representative examples of DNA origami technology. Reproduced with permission [8]. DNA origami structures have been designed with the help of computer programs (Reproduced with permission [14])

The concept of structural DNA nanotechnology was introduced firstly by Nadrian Seeman in the 1980s. He, who is the greatest pioneer in the field, has realized that DNAs can be identified to be a new biopolymer designable in an accurate nano-level [11, 12]. Here, DNA is only appreciated to be a tinker-toy material assembled by some nanotechnologists (Fig. 2a). While three or more strands of DNAs are combined via a thermal fluctuation, a junctional motif is formed among three different strands. Owing to their topological properties, the synthetic DNA constructs have been distinguished clearly from natural DNA forms in the formation process [13–17]. Such DNA nanostructures are simply designated as X- or Y-shaped DNAs. They hold a dimensional structure with single stranded overhangs at the end. The end-overhangs vary greatly with their sequential designs, and they can be equipped with some functional modules through a chemical or enzymatic modification. It then enables to some practical applications involving a pathogenic biomolecular detection [13, 33–35]. Also, together with the end-to-end mutual binding of DNA building blocks in response to specific cue signals, new hydrogel forms composed of only DNAs have been produced [36, 37].

When a long single-stranded RNA fragment originated from natural viruses or bacteria is used as a scaffolding platform and then can react with staple DNA strands which are shorter in length and complementarily hybridized, a large-scaled sheet-like DNA assembled construct can be completed; it is simply referred to as a DNA origami [18, 19, 23–27]. The technique allows the formation of any desired and synthetic DNA structures. With the rapid development of several computer programs, it has aided their design process, simultaneously achieving a final product successfully as designed (Fig. 2b) [38, 39]. Also, it is conceivable to synthesize plenty of structural DNA materials in different dimensions by only altering the portion edges of DNA origami [40]. Such DNA origami has been used remarkably as a guiding template for making a lot of nanomaterials precisely aligned. In addition to the assemblies, it also exhibits some characteristic features such as an enhanced intracellular transport because of its intrinsic biocompatibility and higher cellular uptake efficiency [41]. Also, when structural DNA nanomaterials are used in vivo, they are easily recognized in the immune system depending on the specific sequence and degree of deformation in the substances. Recently, we and our colleagues have completed many experiments to demonstrate the interaction between DNA nanostructure and immune system more specifically. A series of immune responses were convined to be due to the specificity of the shape or the nature of the internal sequence of DNA nanomaterials [42, 43]. With inspiration, we are recently designing a new DNA nanostructure with various complexities of the motifs and are in the process of evaluating their structural/chemical performance. It is believed that these studies will reveal the immunological potential of DNA nanostructures, and expand the usage of DNA nanostructure as a practical therapeutic agent.

### Inorganic compounds

#### Gold

Gold materials, including gold nanoparticles, nanorods, and nanowires, are the most representative inorganic compounds for a variety of biomedical applications [44–46]. The fundamental use of gold nanomaterials in several biomedical issues has mainly been centered onto a photothermal conversion through a surface plasmon resonance phenomenon. Surface oscillations of electrons on the gold nanomaterial lead to a specific absorption spectrum, and a discharge of specific absorbed energy is in a kind form of heat [47]. The optical properties of gold nanomaterial frequently described anywhere are possibly predictable using a finite element analysis method [48, 49]. Especially, an irradiation of gold nanorods by near-infrared lights are commonly used in in vivo photothermal therapy [50]. Moreover, easy fabrication and higher fidelity of the particles integrated facilitates their usage in various researches. For instance, gold nanoparticles can be made facile by a citrate-based fabrication procedure [51]. It is made possible to obtain some nanoparticles in a discrete form, which show higher colloidal stability without additional use of other surfactants. Likewise, gold nanorods can be also produced in the presence of CTAB(Cetyl trimethylammonium bromide) under a harsh redox condition [52]. A seed-mediated fabrication procedure, which is mostly popular in gold synthesis protocol, is quite reproducible with higher reliability in miscellaneous conditions. Gold nanowires could be prepared in a similar procedure proposed for the gold nanorod fabrication [53]. Additionally, several procedures have been introduced to prepare a type of gold nanomaterials possessing various selective shapes, such as nanostars, plates, shells, and cages. More than those, specific sequential DNA assemblies has been also used as a scaffold for producing a variety of gold nanostructures with inimitable and innovative shapes [54].

#### Iron oxide

A magnetic field is a very attractive energy foundation for numerous applications. Magnetic fields can penetrate much deeper into tissue than a near-infrared light, and they do not induce local tissue damage simultaneously [55]. A oxide derivative of Fe or Ni ions in their particle forms below a critical size can induce a explicit superparamagnetism [56, 57]. Such materials are widely used for both deep tissue imaging and therapeutics [57–59].

Iron oxide nanoparticles with a magnetic feature can be prepared by a variety of synthesis protocols, including a coprecipitation or a microemulsion [57]. However, for many years, it was difficult to obtain them massively while maintaining their greater product quality, stability, as well as integrity. In 2004, Park et al. [60] have reported a new thermal decomposition method for the fabrication of highly discrete iron oxide nanoparticles in a special organic solvent. The fabrication procedure has started stepwise with an initial foundation of iron oleate, consecutively followed with its aging at higher temperatures (above 300 °C). It finally allowed a synthesis of iron oxide nanoparticles. Massive amounts of nanoparticles were finally yielded with higher fidelity by following up the simple procedure. The particles formed have possessed a characteristic of magnetic responses, and their additional surface modification allowed the possibility of numerous applications including cancer therapy, catalysis, and magnetic separation which were not dreamed of in the past. In particular, it is increasingly of interest to replace a prevailing and conventional MRI contrast agent [58, 61].

#### Quantum dot

A representative fluorescence-emitting inorganic compound is a quantum dot (QD). The band gap of QDs is adjustable by varying their size, thus allowing numerous alterations in their emission lights ranging from infrared to ultraviolet spectral regions [62]. Other features of quantum dot include a lower photobleaching and higher surface functionality. Early on, Cd-based QDs were commonly applied into several bioimaging areas. In a remarkable achievement were one non-blinking QDs [63]. It has been devised to address and overcome the serious limitations of photo-blinking phenomena, whereby QDs emit a light in a regular period despite an interference of continuous stimulation. Once the emitted light is needed continuously, it is lastly exposed in an extreme damage; Alternatively, Cadmium Selenide (CdSe)/Cadmium Sulfide(CdS) core–shell QDs having no effects on photoblink were developed to solve this problem skillfully. Also, surface coating technology has been introduced and developed to increase its fluorescence stability and to enhance its biocompatibility in a body. Several strategies for amending its surface with some biopolymeric molecules such as poly(lactic-co-glycolic acid) (PLGA) and phospholipids have been proposed, then showing its own enhanced biocompatibility and long-term circulation. It has facilitated several biological applications as a Near-infrared (NIR)-fluorescent agent alternative for some in vivo imaging [64]. For another applications, QDs have been labeled with some biomarkers (e.g. peptide aptamers) and it allowed their specific and selective reactivities to a specific type of cells. In this manner, a business of a cell tracking as well as a targeted cell imaging (e.g. stem cells, cancer cells) could be accomplished lastly [65, 66].

#### Upconversion nanoparticle

A upconversion nanoparticle is a new type of fluorescent nanomaterials recommended lately for biological applications [67]. They have some unique optical properties to absorb a longer wavelength (low-energy) light, then emitting it as a relatively shorter wavelength (high-energy) light. Primarily, trivalent rare earth lanthanide ions (Ln^3+^) are used as a dopant in a specific type of nanocrystals in order to produce exclusive upconversion luminescence properties in nanoparticles. A variety of synthesis methods including a thermal decomposition, a hydrothermal, and a co-precipitation method have been suggested to obtain some discrete upconversion nanoparticle [68]. Unlike the quantum dots presented formerly, upconversion nanoparticles have resulted in the photo-blink, and thus are very feasible for longer-term applications and quantitative analysis. For instance, the long-term visualization of transportation of upconversion nanoparticles in a lymphatic system has been studied recently. Their biocompatibility and stability have been tested in some mice model by observing the movement of mice behavior until upconversion particles were lastly ejected from the test body samples via a sentinel lymph node [69]. In addition to the imaging tool, upconversion nanoparticles have been employed for uses of some therapeutic issues. Especially, for a photodynamic cancer therapy, upconversion nanoparticles have converted NIR lights into visible lights, which activate a photosensitizer adsorbed onto their surfaces and successively generate a reactive oxygen species for the achievement of effective cancer killing [70]. It should be noted that all photosensitizers are excited by either visible or UV light, which showed a limited tissue penetration in depth. Currently, upconversion nanoparticles are a versatile material for a long-term in vivo cell tracking, a verification of therapeutic efficacy, and a cancer treatment.

#### Carbon nanotube

A carbon nanotube is a graphite sheet that is grounded into a nanoscale diameter. Depending on its surface’s angle and nanostructure, the carbon nanotube exhibits a special character of a metallic or semiconductor [71]. According to the wall number, it can be classified into a single-walled carbon nanotube, a double-walled carbon nanotube, a multiwalled carbon nanotube, and a rope-typed carbon nanotube. They have lots of unusual physiochemical properties and can be also used as a main resource in several research fields involving a nanotechnology, an electrical engineering, an optical engineering, and a materials engineering. Owing to their intrinsic fluorescence characteristics in the second biological window NIR-II (1000–1700 nm) upon excitation in the traditional NIR-I region (700–950 nm), they are used as a promising nanomaterial in several biomedical applications [72]. The NIR-II region provides a reduced tissue scattering and an auto-fluorescence, still by reaching a deeper penetration in depth of in vivo, as compared to a traditional NIR-I imaging technique [73]. Mouse brain imaging is greatly achievable using such carbon nanotubes. Their photoluminescence in 1300–1400 nm NIR regions have allowed to reach a depth of more than 2 mm for much clearer bioimaging. Recently, some mouse brain imaging was done successfully up to 3 mm in depth using carbon nanotubes revealing the photoluminescence in the 1500–1700 nm NIR region [74].

## Integration of inorganic compounds with structural DNAs; structural DNAs-inorganic hybrid complexation and their practical applications

In this section, we will look over some cases regarding a complexation of new hybrid materials and their practical applications. Here it is described in more detail that structural DNA nanomaterials have been integrated together with inorganic compounds described previously and how they are available, with working on their synergetic effects, for some practical applications including biomedicines for disease diagnosis and treatments. We here have excluded some cases showing the uses of conventional linear or circular DNAs as a probing moiety, once combined with inorganic nanoparticles.

### Gold-DNA complex

**Fig. 3:**
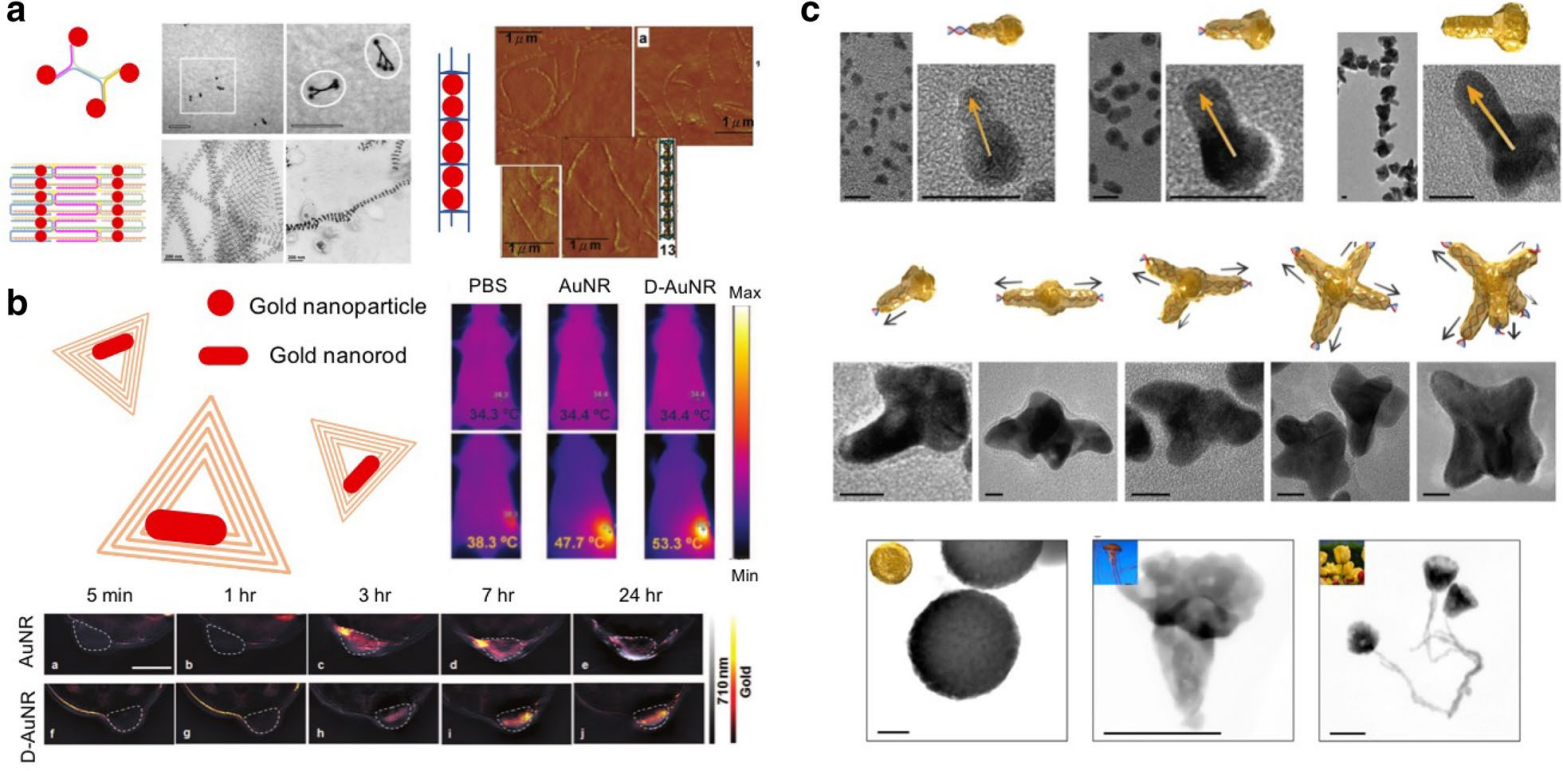
**a** Alignment of gold nanoparticles using a variety of DNA nanostructures. Branched DNA nanomaterials [44], DNA tiles [45], and DNA tubes [46] were used for the ordered alignment. Reproduced with permission. **b** Targeted delivery of gold nanorods using triangular DNA origami. Targeted delivery has enabled an optoacoustic imaging and a photothermal therapy. Reproduced with permission [48]. **c** Growth of gold nanomaterials guided by structural DNA nanomaterials (Reproduced with permission [49])

As discussed earlier, gold nanomaterial is to absorb a light, followed by transferring its energy into heat. Its photodynamic capability has been utilized well into the use of photothermal therapy and even optoacoustic imaging. Attaching single-stranded DNAs as a detection probe on the surface of gold nanoparticles and then inducing a variation of the plasmon coupling or light absorption spectrum upon interaction with target DNAs is known to be one of the common strategies. In addition, there have been new-fangled attempts to put the gold nanoparticles in an ordered array using simple branched DNA nanotechnologies [75], DNA origami [76, 77], and DNA tube (Fig. 3a) [78]. Especially, the uses of DNA origami have allowed a precise positioning of gold nanorods, with their surface plasmon resonance coupling effects controlled in an efficient way [79, 80].

Despite the exceptional and greater functionalities of gold nanomaterials, it is much challenging to deliver some therapeutic gold nanomaterials to target cell sites in an accurate way. Structural DNA nanomaterials are a good candidate for revising such downsides and exploiting its performance better. For instance, DNA nanostructures, especially a triangular DNA origami, has tolerated an optimized tumor cell targeting without any initiation of systemic toxicity [41]. Yang Du et al. have also developed very exceptional optoacoustic imaging agent combining two different features of gold nanomaterial and DNA origami in a synergetic manner (Fig. 3b) [81, 82]. In their study, a M13mp18 genomic DNA strand was used as a template for DNA origami, and a capture strand extending from the origami was hybridized specifically with complementary strands embedded on the surface of gold nanorods. Through a thermal annealing process, gold nanorods were simply placed at the specific binding site of the DNA origami and then assembled into an ordered pattern. Most interestingly, the hybrid complexes of gold nanorods and DNA origami acted as very promising contrast agent for the optoacoustic imaging, enabling an improved resolution quality even with reduced doses concerned primarily in in vivo application. At the same time, the hybrid complexes acted as a photothermal therapy agent, effectively inhibiting a tumor regrowth and prolonging a survival in diseased mice in response to the NIR irradiation.

In views of the hybrid complexation between gold nanomaterial and structural DNA, it is probably achievable to get ready for some gold nanomaterials with a variety of desired shapes by using a structural DNA template (Fig. 3c) [83]. It has been proven with all-atom simulation that highly concentrated with tetracloroaurate (AuCl_4_) and hydroxylammonium chloride (NH_3_OH^+^) ions around a double-stranded DNA template have stimulated some redox reactions, thereby producing a DNA-directed metallization. Thus, a new gold nanomaterial has been formed along the pre-formed morphology of DNA-templated nanostructures. It is also possible to fabricate a singular gold nanowire in a similar manner, and many reports have been published. Of them, there is a nanowire-based electrical sensor which can effectively diagnose specific biomarkers in a body. According to the used DNA strands, the shapes of gold nanomaterials produced can be greatly varied. It thus helped them facilitate their intracellular transport [84].

### Iron oxide-DNA complex

**Fig. 4:**
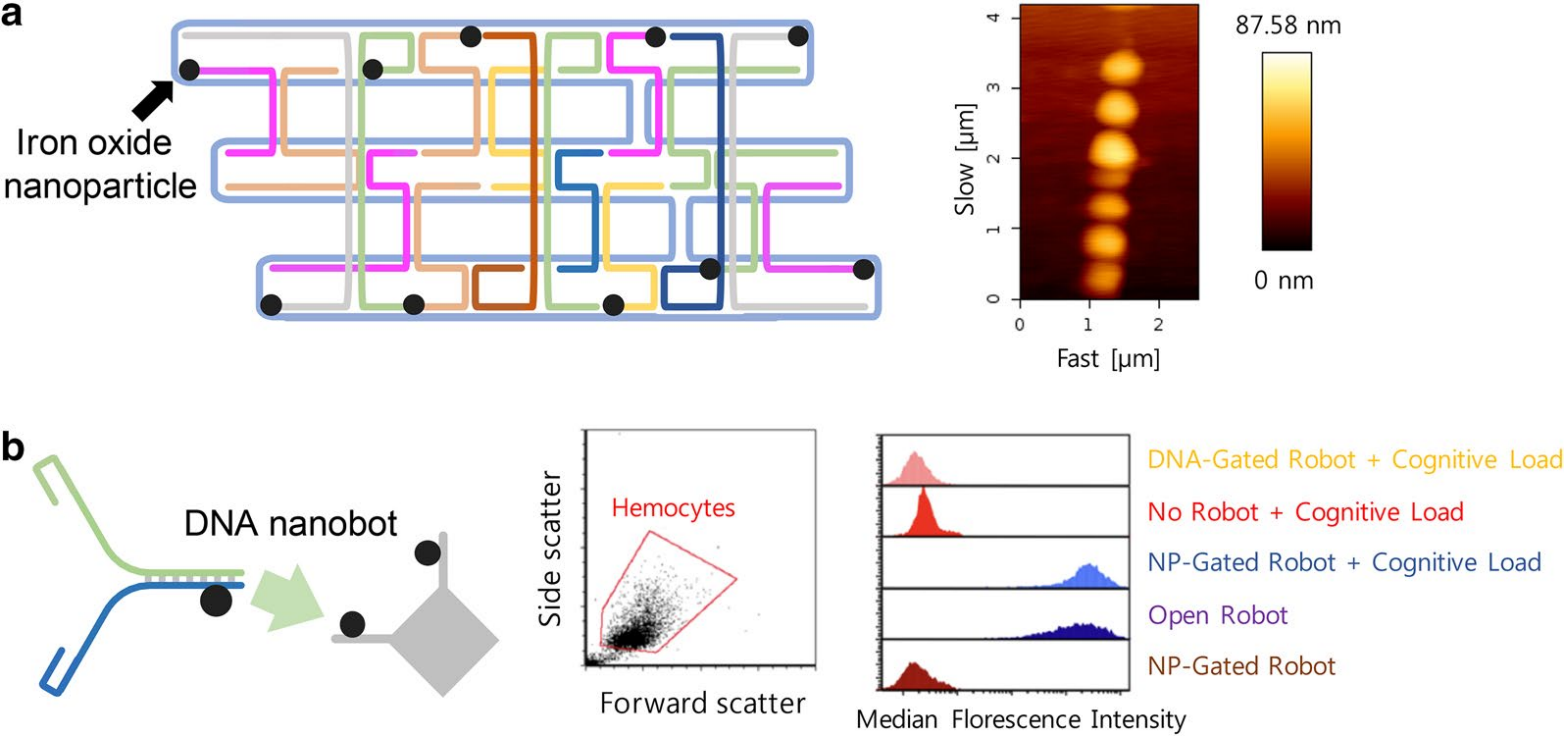
**a** Guided alignment of iron oxide nanoparticles on DNA nanotubes. Iron oxide nanoparticles were attached on the surface of DNA nanotube using a biotin–streptavidin binding chemistry. Reproduced with permission [52]. **b** Iron oxide nanoparticle-loaded DNA nanobot. DNA nanobots have recognized external magnetic fields in the used insects, then producing an in vivo cell effect (Reproduced with permission [53])

Due to the superparamagnetism in magnetic fields, iron oxide nanoparticles have received much attention in several practical applications [85]. For instance, in the field of biopharmaceuticals, it is made possible to isolate some biological substances like genes and proteins using a magnetic separation technology, and achieve an effective in vivo imaging further. The effects of heat generated by the alteration of rapid magnetic fields have been applied into some thermal therapies. As for gold-based hybrid complexations, DNA origami has been exploited to accurately position iron oxide nanoparticles at desired locations [86]. Rafati et al. have fabricated a new DNA nanotube using a M13mp18 phage genome as a scaffold, and it made iron oxide nanoparticles aligned onto the surface of DNA nanotubes using a biotin–streptavidin binding chemistry (Fig. 4a).

There are a few studies in which DNA origami was combined with iron oxide nanoparticles in order to exploit their thermal synergetic characteristics. A system merging DNA nanobots with iron oxide nanoparticles has been proposed (Fig. 4b) [87]. When iron nanoparticles were equipped with DNA nanobots, its internal payload was separated from its nearby environments. The shell’s opening process made the nanoparticle’s payload exposed certainly into neighboring circumstances. Therefore, by controlling the shell’s shuttering precisely, some therapeutic molecules placed inside have been reversibly switched on and off in either exposure or concealment states, and a thermal energy generated temporarily by iron oxide nanoparticles was explored in accordance with the fluctuations of the given magnetic fields. Arnon et al. have succeeded in recognizing the activation of DNA robots by stimulating a cell effect inside an insect named as *Blaberus discoidalis*. It is highly expected that such technology may be a new disease treatment modality for a few of serious disease disorders such as schizophrenia, depression, which may be generally thought of some of the most difficult ones to rectify.

### Quantum dot-DNA complex

QDs are a nanomaterial whose fluorescence characteristics can be manipulated effectively according to their size, and they are highly applicable into several bioapplications including a fluorescence cytometry. Analogous to similar nanomaterials mentioned once, there have been many studies in which DNA origami was used as a template to position a QD in a desired position [88–91]. In some case, the QDs were synthesized using a nucleic acid sequential template [92]. The DNA sequential domains are primarily composed of three portions: a phosphorothioate backbone, a spacer, and a phosphate bond. The phosphorothioate backbone is strongly bound, with the highest value of dissociation coefficients, to the cation of QDs. Such differential affinity may have them attached selectively to QDs. The QD-DNA hybrid complexes, sometimes with their overhangs freely opened, have been assembled into a 3-dimensional DNA hydrogel form. QD-DNA hybrid hydrogels were also used as a drug-loaded vehicle, thus increasing its toxic potency against targeted cancer cells up to ninefolds.

**Fig. 5:**
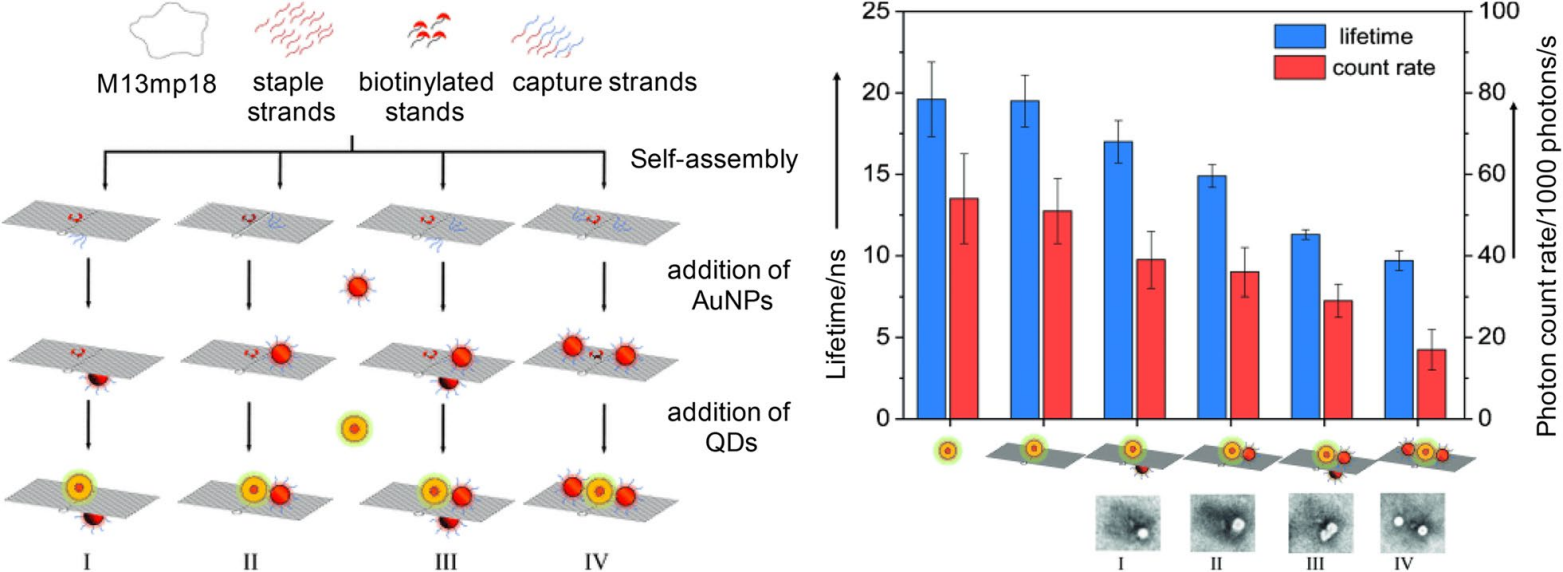
Precise positioning of both gold nanoparticles and QDs, once being templated with DNA origami. Improved optical properties, including a colorimetric and fluorescence quantification and lifetime, were controlled by the relative distance and location of gold nanoparticles and quantum dots (Reproduced with permission [57])

It has also been proven that a fluorescence lifetime of QDs can be controlled by placing together quantum dot and gold nanoparticle on the surface of DNA origami, efficiently adjusting their relative positions [93]. Ko et al. have used a triangular DNA origami to precisely put gold and QD nanoparticles at a certain and desired location (Fig. 5). In some cases, both gold nanoparticles and quantum dots have been arranged in a cubic DNA nanostructure as a quencher and a fluorescent molecule, respectively [94]. It allowed a simultaneous colorimetric and fluorescence quantification of two different molecules.

### Upconversion nanoparticle-DNA complex

**Fig. 6:**
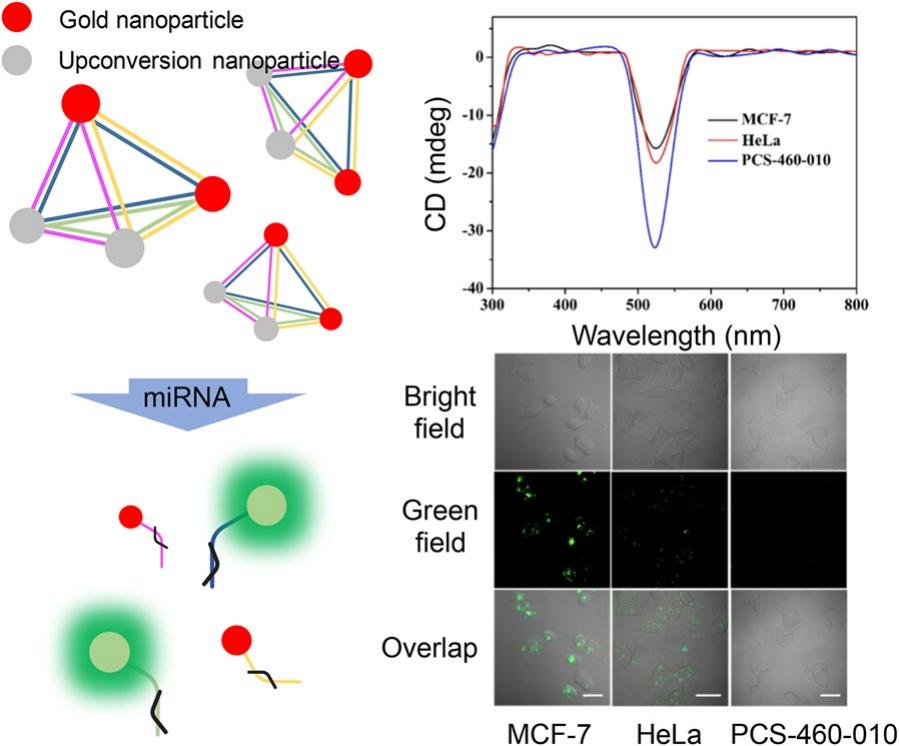
DNA pyramid structures including gold nanoparticles and upconversion nanoparticles. The DNA pyramid disintegrates its original structures upon binding specially to intracellular miRNAs, thus restoring suppressed optical properties of the upconversion nanoparticles involved in the system (Reproduced with permission [58])

Due to their intrinsic optical properties, NIR light, anti-Stokes emission, excellent light stability and chemical stability, lower toxicity and higher signal-to-noise ratio, upconversion nanoparticles have many promises in several practical applications. It is still in its infancy and relatively little research has been done. In the past, it was a study to recognize dna targets using excellent diagnostic ability of Upconversion particles [95, 96], but recently it has been focused on the formation of composites through deformation of dna using structural nucleic acid engineering [97]. A variety of applications have been made possible by being combined with some structural DNA nanostructures, which provide a regular geometry and structural stability at a nanoscale level. It included the utilization of DNA pyramids containing a gold nanoparticle and an upconversion nanoparticle to detect targeted mitochondrial RNAs in a cell (Fig. 6) [98]. The unusual structure of DNA pyramid has extinguished the emission of upconversion nanoparticles by allowing a luminescence resonance energy transfer between gold nanoparticle and upconversion nanoparticle. The presence of the targeted miRNAs caused the DNA pyramids to break down, thus separating upconversion nanoparticles from gold nanoparticles. The pyramid complex enabled a detection of miRNAs in a cell by means of its strong plasmonic circular dichroism and a significant luminescence. In the presence of targeted miRNAs, the circular dichroism’s signals have been decreased while its luminescence intensity simultaneously increased, providing an extremely sensitive and highly selective detection of miRNAs in a living cell, based on their dual-mode signaling. Although it is not easy to see the case yet, many related studies are being planned and underway due to the stimulation of the efforts of Kotov and Xu presented above.

### Carbon nanotube-DNA complex

**Fig. 7:**
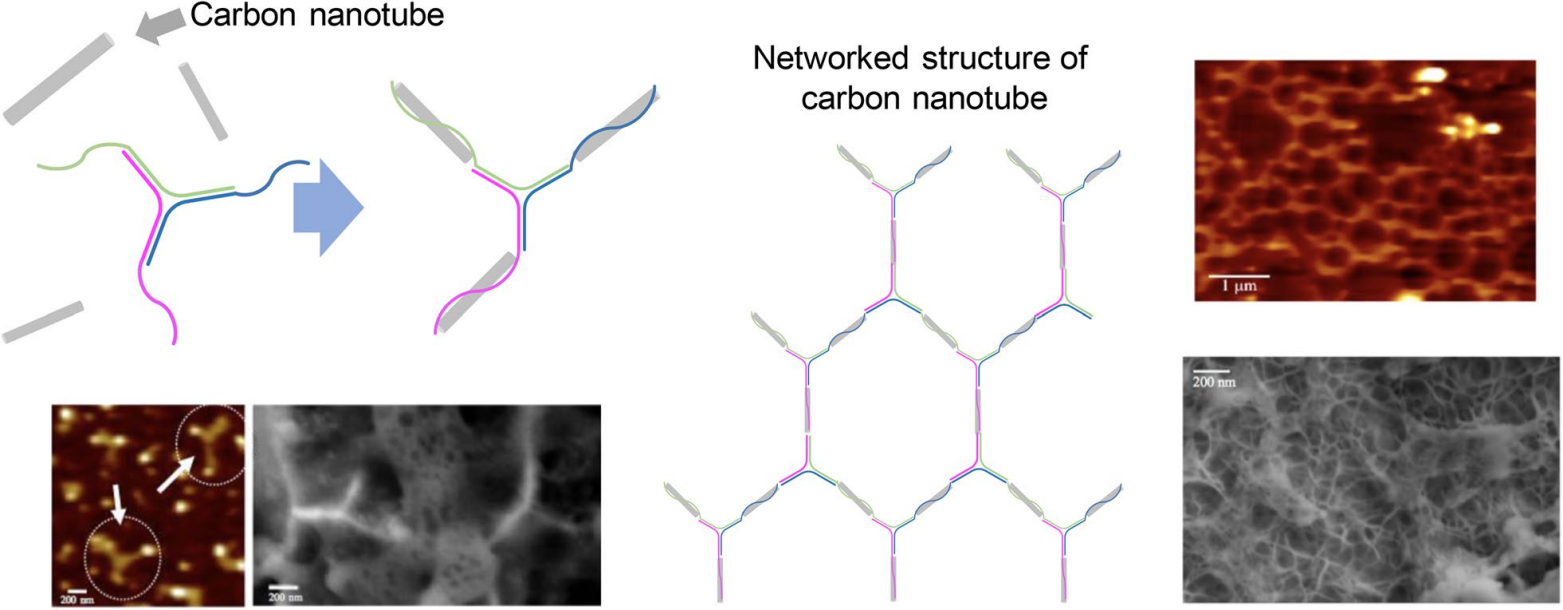
Guided alignment of carbon nanotubes using a Y-shaped DNA nanomaterial. Selective binding of carbon nanotubes was made possible with poly(G) sequences wrapping the nanotubes. A network of carbon nanotubes was formed with repetitive Y-shaped DNA nanomaterials (Reproduced with permission [60])

The electrical and optical properties of carbon nanotube have made it possible to apply the precisely aligned carbon nanotube assemblies into a sensor as well as a transistor. Based on the stronger π–π interactions between single-stranded DNA and carbon nanotube, DNA strands tend to form a spiral wrapping around the carbon nanotubes [99], and this outcome can serve as a useful tool kit for a distribution, classification, and tagging of carbon nanotubes. Recently, carbon nanotubes were greatly aligned by simply combining these with structural DNA nanostructures [100]. Oruc et al. have used a Y-shaped DNA structure having three different arms for the carbon nanotube alignment, and some binding among them was made possible by adding a poly(G) sequence into the end overhang of Y-DNAs. The orientation of three carbon nanotubes at the angle of about 120° was confirmed obviously by a scanning electron microscopy and an atomic force microscopy (Fig. 7).

Controlled assembly of carbon nanotubes has been also executed [101–103]. Inspired by the accurate positioning of carbon nanotubes on a two-dimensional DNA origami, it was possible to design a universal set of carbon nanotube circuit [104]. Czeizler et al. have fabricated a set of 14 differentiated DNA origami tiles, each with one carbon nanotube—based electrical subunit being positioned to its surface. By tempting some DNA tiles containing each subunit to self-assemble, it is made possible to manufacture more complex and integrated circuits.

Besides the positioning of nanotubes on the DNA structures, such complexes are beginning to be applied to new other attempts including bioapplications. For instance, due to its excellent cell-friendly properties such as the robustness of nanotubes and high cell permeability, dna or dna structures are increasing intracellular permeability with the help of nanotubes [105]. Or DNA nanostructures that simulate the characteristics of nanotubes are designed and replaced [106]. Recent advances in these carbon nanotube-DNA complexes have begun to realize this potential but is still at the earliest stage and a number of hurdles remain.

## Conclusion and perspective

In this review, we have examined how structured DNA nanotechnology was cooperated with inorganic compounds, further showing many practical applications using their hybrid complexes. Gold, iron oxide, quantum dot, and other representative inorganic nanomaterials integrated strategically with structural DNAs have been created, and their synergetic improvements in functionality have been highlighted in some ground-breaking applications. Many outstanding studies on the hybrid material of structural DNA and inorganics are confirming that they have numerous advantages over conservative material resources which, sometimes, exist in each single form. Structural DNA nanomaterials as a template have enabled a precise positioning and ordered alignment of inorganic nanomaterials and, occasionally, they have been proven further to sometimes enhance or lessen characteristic properties for progressive applications that was not dreamed of in the past. It has been established that, for instance, they regulated the optical properties for progressive bioimaging and improved some intracellular permeability and thereby allowed an efficient transmission to infected disease sites for practical uses as a therapeutic agent as well as an exploitation of improved optical properties.

However, it is still in early stage and few relevant systems have been practically in use. Hopefully, it is gradually being replaced with a realistic alternative beyond the limitations of existing systems, and soon we will live and evolve in a world that is driven by these new materials [107].

## Data Availability

Not applicable.
